# The role of sortilin in cardiovascular calcification: mechanisms and therapeutic potential

**DOI:** 10.3389/fcvm.2026.1805742

**Published:** 2026-06-10

**Authors:** Yaqing Yao, Sijia Li, Shuangshuang Wang

**Affiliations:** 1 Department of Cardiology, Wenling Hospital of Wenzhou Medical University (The First People’s Hospital of Wenling), Wenling, Zhejiang, China; 2 Psychology Department, Australian National University, Canberra, ACT, Australia

**Keywords:** aortic valve calcification, risk factors, sortilin, therapeutic target, vascular calcification

## Abstract

Cardiovascular calcification is a common pathological feature driving cardiovascular morbidity and mortality. Although traditionally viewed as a passive degenerative process, current evidence demonstrates that calcification is an actively regulated, cell-driven phenomenon involving osteogenic differentiation, inflammation, and metabolic disturbances. Sortilin (encoded by *SORT1*), a multi-ligand intracellular trafficking receptor, has recently emerged as a critical molecular mediator linking metabolic risk factors—such as dyslipidemia, diabetes, hypertension, and chronic kidney disease—to pro-calcific cellular responses. In addition, sortilin also plays a versatile role by directly participating in the regulation of the phenotypic transformation of vascular smooth muscle cells and valve interstitial cells. This review provides a comprehensive synthesis of SORT1 biology in calcification-related risk states, delineates mechanistic insights into its specific roles in vascular and valve tissues, and discusses translational opportunities and challenges for targeting SORT1 in the prevention and treatment of cardiovascular calcification.

## Introduction

Cardiovascular calcification refers to the pathological deposition of calcium within the cardiovascular system, manifesting as vascular calcification, aortic valve calcification, and other localized mineralization processes. It significantly contributes to cardiovascular mortality among patients with diabetes, atherosclerosis, chronic kidney disease (CKD), and hypertension (1). Despite its clinical relevance, no pharmacological therapy has yet demonstrated the ability to halt or reverse calcification. Rather than a passive degenerative outcome, cardiovascular calcification is now recognized as an active, tightly regulated process driven by complex cellular and molecular events (2). Comprehensive investigation into the mechanisms governing its initiation and progression is essential for the development of effective therapeutic strategies.

The *SORT1* gene, located on chromosome 1p13.3, encodes sortilin, a type I sorting receptor localized within intracellular compartments—such as the endoplasmic reticulum, Golgi apparatus, lysosomes—as well as on the cell surface (3). Sortilin functions as a trafficking hub for diverse extracellular ligands and intracellular cargo, regulating lipid and glucose metabolism, extracellular vesicle biogenesis, autophagy, and cell survival (4, 5). Large-scale genetic studies have linked the *SORT1* locus to coronary artery calcification, early-onset myocardial infarction, and abdominal aortic aneurysm (6). Moreover, sortilin modulates LDL metabolism, VLDL secretion, PCSK9 trafficking, and cytokine release, thereby influencing multiple components of atherogenesis (7, 8).

Sortilin has attracted considerable scientific interest in recent years for its significant involvement in cardiovascular and metabolic disorders, positioning it as both a viable biomarker and a potential therapeutic target. Although early SORT1 research primarily addressed non-calcific cardiovascular mechanisms, emerging data now underscore its relevance to cardiovascular calcification. This review aims to delineate the role of SORT1 by evaluating recent findings that connect SORT1 to cardiovascular calcification, and concluding with a synthesis of pathophysiological insights and therapeutic implications.

## Sortilin and calcification-related risk factors

Cardiovascular calcification is a progressive and clinically consequential process that results from a complex interaction of traditional and non-traditional risk factors. Advanced age and male sex represent well-established non-modifiable demographic risks (9). Key modifiable clinical contributors include chronic kidney disease (CKD), diabetes mellitus, dyslipidemia, and hypertension (10). Growing evidence highlights sortilin as a salient molecular node that integrates several of these pathogenic pathways. Thus, SORT1 appears to function as a convergent signaling hub that transduces diverse risk-factor stimuli into pro-calcific responses, providing a potential mechanistic framework for understanding individual susceptibility and informing future therapeutic strategies.

### Sortilin and hypertension

zHypertension, a leading cause of global mortality with increasing incidence among adults, is often asymptomatic yet poses a significant long-term risk for cardiovascular calcification. Clinically, circulating sortilin levels are associated with essential hypertension. A cross-sectional study found that elevated plasma sortilin is independently linked to the presence of hypertension and subclinical carotid atherosclerosis (11). This aligns with research indicating that soluble sortilin, released by activated platelets, has circulating levels that correlate with cardiovascular risk factors, including those related to hypertension (12). Consequently, sortilin is being investigated for its diagnostic utility, serving as a potential biomarker for identifying atherosclerosis in individuals with hypertension (13). SORT1 has been confirmed to contribute to the development of hypertension through direct regulation of vascular function (14). Sortilin promotes hypertension by disrupting sphingolipid/ceramide homeostasis and inducing oxidative stress within the vasculature (15). Furthermore, its pathogenic effect involves the activation of the acid sphingomyelinase (ASMase)/sphingosine-1-phosphate (S1P) axis, and targeting this pathway has been shown to protect against sortilin-evoked vascular damage in hypertension models (16). Together, these findings position sortilin not only as a key molecular player in hypertensive vascular remodeling and damage but also as a promising biomarker and potential therapeutic target for hypertension-related cardiovascular complications.

### Sortilin and dyslipidemia

Dyslipidemia is a central driver of atherosclerosis and also provides a critical pathological basis for cardiovascular calcification. Elevated levels of low-density lipoprotein cholesterol (LDL-C), particularly small, dense LDL particles, are susceptible to oxidative modification. Oxidized LDL (ox-LDL) contributes to endothelial dysfunction, promotes chronic inflammation and oxidative stress, and thereby initiates cardiovascular calcification (8). Other dyslipidemic phenotypes, such as hypertriglyceridemia and low high-density lipoprotein cholesterol (HDL-C), further exacerbate calcification by modulating inflammatory responses and cellular functions (17, 18). Thus, dyslipidemia constitutes a crucial intermediate link connecting metabolic dysregulation to calcification and serves as an indispensable pathogenic factor in the initiation and progression of cardiovascular calcification.

Sortilin plays a well-established but complex and sometimes contradictory role in dyslipidemia pathogenesis, supported by human genetics, clinical biochemistry, and experimental models. Genome-wide association studies (GWAS) have repeatedly identified the SORT1 locus at 1p13.3 as one of the strongest signals associated with plasma LDL-C levels and coronary artery disease (CAD) risk (19, 20). The minor allele at this locus is linked to lower LDL-C and reduced CAD risk (21). Elevated circulating sortilin levels are observed in patients with CAD and correlate positively with LDL-C, triglycerides, and PCSK9, supporting its potential as a cardiovascular risk biomarker (12, 22).

Mechanistically, sortilin functions as a multi-ligand intracellular trafficking receptor (Vps10p-domain family) that modulates lipoprotein metabolism at multiple levels, primarily in hepatocytes (20). However, its net effect on plasma lipids has been the subject of significant debate, with studies reporting apparently opposing outcomes depending on experimental context, model system, and metabolic state. Several lines of evidence indicate that sortilin raises circulating LDL-C under most conditions. Sortilin directly binds apoB100 in the Golgi apparatus, facilitating the formation and export of apoB100-containing VLDL particles (20, 23). Consistent with this, hepatocyte-specific Sort1 knockout significantly reduced plasma cholesterol, attenuated hepatic VLDL secretion, and alleviated hypercholesterolemia (24). Human genetic evidence, including missense variants in SORT1 (e.g., K302E), further supports that higher sortilin activity increases LDL-C (25). Additionally, sortilin enhances the secretion of apolipoprotein(a), thereby promoting Lp(a) levels (26).

In contrast, under obese, insulin-resistant, or ER-stressed conditions, hepatic sortilin expression is markedly downregulated, which paradoxically contributes to dysregulated VLDL overproduction. In Western diet-induced obese and diabetic mice, activation of mTORC1 and ER stress strongly represses hepatic SORT1, leading to increased VLDL secretion and hyperlipidemia (27, 28). In this pathophysiologic context, restoration or overexpression of sortilin improves lipid profiles by reducing VLDL export and lowering plasma cholesterol (29). Thus, increasing sortilin appears beneficial specifically when its levels have been suppressed by chronic metabolic stress.

These apparent discrepancies reflect clear context-dependent effects of sortilin. In normolipidemic or mildly dyslipidemic states, sortilin acts primarily as a pro-lipogenic trafficking receptor; therefore, knockdown or loss-of-function reduces VLDL secretion and lowers blood lipids. However, in established obesity, type 2 diabetes, or chronic ER stress, hepatic sortilin is already pathologically downregulated. Further knockdown may have limited additional effect or even be neutral, whereas restoring sortilin to normal levels corrects excessive VLDL production, possibly by re-establishing proper apoB100 sorting/degradation balance and improving hepatic insulin signaling. Species differences, tissue specificity (hepatocyte vs. macrophage vs. platelet-derived soluble sortilin), and the multi-ligand nature of sortilin further complicate the picture.

In summary, sortilin is a pivotal genetic and functional regulator of lipoprotein metabolism whose net impact on dyslipidemia integrates hepatic VLDL production, receptor trafficking, and systemic inflammation. The conflicting data highlight the need for nuanced, context-specific therapeutic strategies—such as inhibiting sortilin in early dyslipidemia vs. restoring it in obesity-associated metabolic stress—when considering SORT1 as a target for treating dyslipidemia and cardiovascular calcification.

### Sortilin and diabetes mellitus

Diabetes is a major independent risk factor for both the initiation and progression of cardiovascular calcification, engaging in a multifactorial interplay with calcification pathways (30). Hyperglycemia and insulin resistance are central to this link. Chronic hyperglycemia drives the formation of advanced glycation end products (AGEs) and activates the RAGE pathway, which directly stimulates cardiovascular cells to adopt an osteogenic phenotype (31, 32). It also amplifies oxidative stress and chronic inflammation, fostering a microenvironment that promotes abnormal calcium deposition. Furthermore, insulin resistance and the resulting compensatory hyperinsulinemia not only directly contribute to cardiovascular calcification but also interact synergistically with dyslipidemia, mineral metabolism disturbances, and adipokine dysregulation, collectively accelerating the calcification process (8, 33). Therefore, diabetes acts as a critical “metabolic amplifier,” significantly elevating the risk of cardiovascular calcification and driving its progression through a network of interconnected pathological signals.

The role of sortilin in diabetes is supported by evidence ranging from genetic associations and clinical biomarkers to defined molecular mechanisms. Circulating sortilin levels are elevated in patients with type 2 diabetes and correlate with the presence and severity of diabetic complications, such as peripheral artery disease and coronary atherosclerosis (34, 35). In diabetic patients with chronic limb-threatening ischemia, higher baseline serum sortilin levels are predictive of major adverse cardiovascular and limb events following revascularization (36). Furthermore, in gestational diabetes mellitus, maternal serum sortilin levels are significantly increased and correlate with insulin resistance indices, suggesting its potential as a novel biomarker (37).

The pathophysiological link between sortilin and diabetes is gaining increasing attention due to its complex role in regulating glucose metabolism. It is essential for the formation and intracellular retention of GLUT4 storage vesicles in adipocytes, thereby regulating insulin-stimulated glucose uptake (38). In insulin-resistant states, such as obesity and type 2 diabetes, the expression of sortilin and related trafficking proteins (e.g., sorting nexins) is reduced in skeletal muscle, contributing to impaired GLUT4 translocation and systemic insulin resistance (39, 40). Additionally, sortilin interacts with neurotensin and its receptors, modulating pathways involved in fat absorption, obesity, and insulin resistance (41). In the diabetic retina, sortilin upregulation contributes to neurodegeneration, while its inhibition provides neuroprotection (42). In pancreatic beta-cells, sortilin-derived peptides promote cell survival via the CREB signaling pathway, highlighting a protective role (43). Additionally, insulin resistance is a major contributor to the hepatic overproduction of APOB100 and triglycerides. Recent research further reveals that SORT1 plays a key role in this pathway by modulating the metabolism of hepatic apoB100 under insulin-resistant conditions (44). Moreover, in diet-induced obesity models, SORT1-deficient mice exhibit reduced weight gain and demonstrate enhanced glucose uptake during insulin tolerance tests (45), suggesting that SORT1 regulates energy balance and glucose metabolism. Thus, SORT1 functions as a critical node connecting metabolic dysregulation and insulin resistance in diabetes.

### Sortilin and CKD

Chronic kidney disease markedly increases the risk of cardiovascular calcification—a key contributor to morbidity and mortality in this population. This elevated risk is attributed to the convergence of traditional factors such as hypertension and diabetes with CKD-specific disturbances in mineral metabolism, chronic inflammation, and uremic toxin accumulation. Emerging evidence underscores a significant link between sortilin and cardiovascular calcification in CKD, positioning it as both a biomarker and a mechanistic contributor. Elevated circulating sortilin levels in CKD patients are independently associated with the severity of vascular and valvular calcification, serving as a promising diagnostic marker (46). Sortilin is also implicated in pathways relevant to CKD-associated cognitive impairment, highlighting its broader role in uremic complications (47). Collectively, these findings position sortilin not only as a potential biomarker for cardiovascular calcification in CKD but also as a plausible mediator driving calcification progression. Further research is warranted to clarify whether modulating sortilin could represent a novel therapeutic strategy to mitigate cardiovascular risk in this vulnerable population.

## The role of sortilin in cardiovascular calcification: vascular and valvular pathogenesis

**Figure 1:**
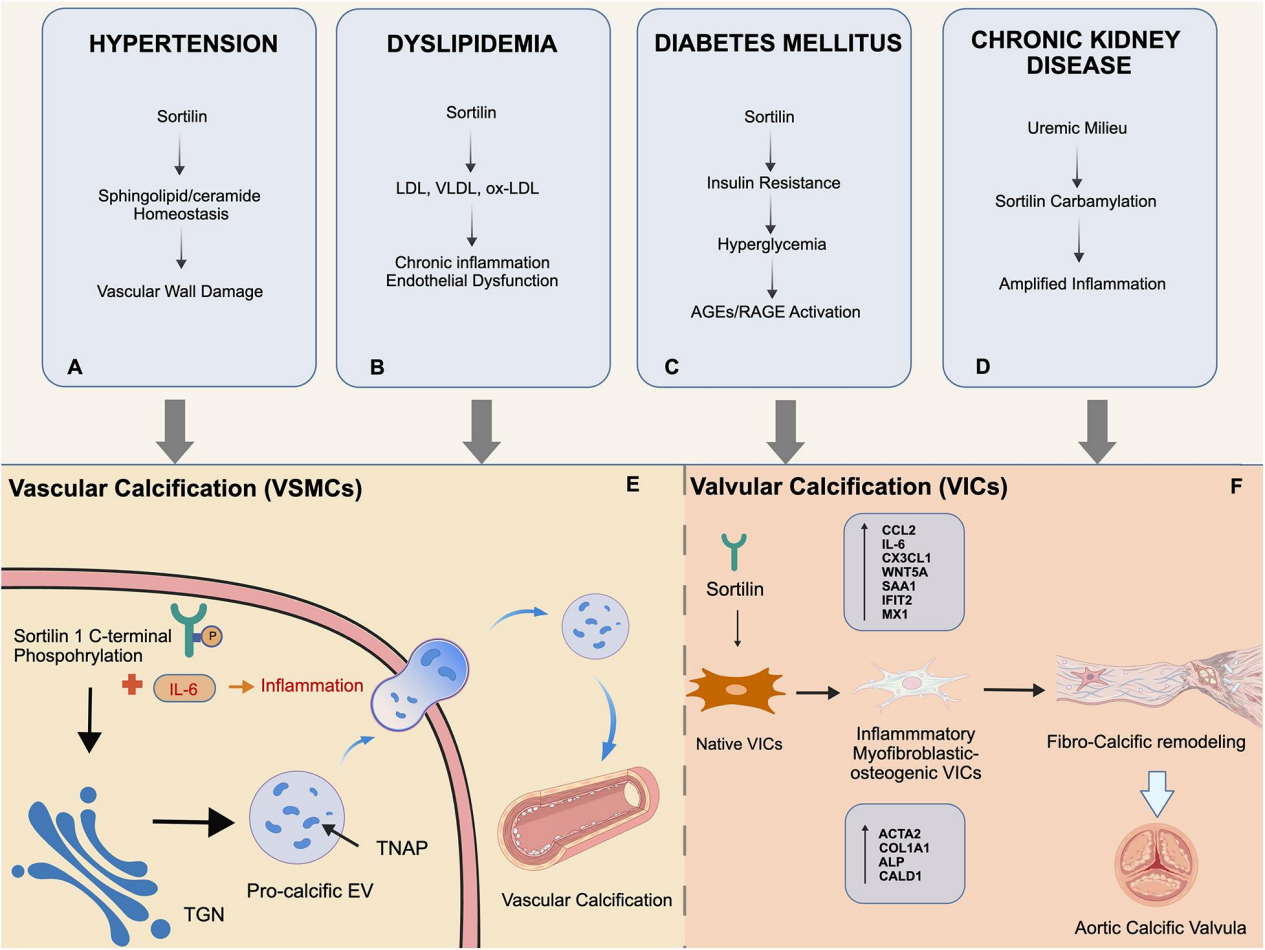
The role of SORT1 in cardiovascular calcification. **(A)** Sortilin promotes hypertension by disrupting sphingolipid/ceramide homeostasis, which leads to vascular wall damage. **(B)** Sortilin modulates low-density lipoprotein (LDL) and very low-density lipoprotein (VLDL) metabolism, increasing plasma oxidized low-density lipoprotein (ox-LDL) levels, which subsequently promotes chronic inflammation and endothelial dysfunction. **(C)** Sortilin contributes to insulin resistance and chronic hyperglycemia, subsequently activating advanced glycation end products and the RAGE signaling pathway. **(D)** The uremic milieu induces sortilin carbamylation, enhancing its binding to interleukin-6 and significantly amplifying inflammatory response. **(E)** Sortilin drives the biogenesis of pro-calcific extracellular vesicles and the specific loading of tissue-nonspecific alkaline phosphatase for secretion. This promotes the osteogenic transformation of vascular smooth muscle cells, ultimately causing the pathological deposition of calcium. **(F)** Sortilin regulates the phenotypic transformation of native valve interstitial cells into myofibroblastic-osteogenic cells. This pathogenic cell population significantly upregulates inflammation-associated molecules including CCL2, IL-6, CX3CL1, WNT5A, SAA1, IFIT2, and MX1, while highly expressing fibrotic and osteogenic markers such as ACTA2, COL1A1, ALP, and CALD1. These pathological molecular alterations directly promote local fibro-calcific remodeling, ultimately resulting in aortic valve calcification (Figure adapted from images created with biogdp.com).

Functioning through the interrelated pathways of vascular and cardiac valve calcification, sortilin has emerged as a critical regulator in cardiovascular calcification pathogenesis. Its role across these mechanisms positions it as a central integrative factor and a promising therapeutic target for related metabolic and ageing-associated disorders (Figure 1).

### Sortilin in vascular calcification

Vascular calcification, particularly within the arterial intima and media, is an active, cell-mediated process resembling osteogenesis, driven primarily by the phenotypic transformation of vascular smooth muscle cells (VSMCs). These cells adopt an osteochondrogenic phenotype, characterized by the downregulation of contractile markers and the secretion of pro-calcific extracellular vesicles (EVs) that serve as nucleation sites for hydroxyapatite crystal deposition. Sortilin is intimately involved in this pathological reprogramming. Clinical and observational studies first established the correlation, demonstrating that elevated circulating sortilin levels are strongly associated with the severity of abdominal aortic and coronary artery calcification and serve as an independent risk factor for major adverse cardiovascular events (46, 48, 49).

The molecular mechanisms underlying this association are now well-defined. Sortilin functions as a key intracellular trafficking receptor in VSMCs, directly driving the formation of calcifying EVs. It facilitates the specific loading of mineralization-promoting cargo, such as tissue-nonspecific alkaline phosphatase (TNAP), into these vesicles for secretion (50, 51). This process is regulated by post-translational modifications; for instance, phosphorylation of SORT1's C-terminal domain is required for its routing to EVs and subsequent promotion of calcification (50). *In vivo* evidence corroborates its causal role, as genetic deficiency of sortilin (*Sort1*^−/−^) significantly reduces atherosclerotic plaque calcification in mice (50, 52). Furthermore, a disease-specific mechanism is highlighted in CKD, where the uremic milieu induces sortilin carbamylation. This modification enhances its affinity for interleukin-6 (IL-6), and the carbamylated form is independently associated with the presence and accelerated progression of coronary artery cacification in patients (53). Notably, the therapeutic potential of inhibiting sortilin may be influenced by sex-specific factors. Experimental studies using antisense oligonucleotides (ASO) against *Sort1* revealed a sex-dependent effect in reducing vascular calcification, with a significant reduction observed in male but not female mice, a discrepancy potentially mediated by β-estradiol's influence on autophagy pathways (52). Therefore, sortilin acts as a central molecular orchestrator in vascular calcification, bridging metabolic stress to the active secretion of calcification-competent EVs from VSMCs.

### Sortilin in aortic valve calcification

The pathogenesis of calcific aortic valve disease (CAVD) involves a complex and dynamic interplay among chronic inflammation, progressive fibrosis, and ectopic calcification (54). Valvular interstitial cells (VICs), the main resident cells, undergo pathological activation towards pro-inflammatory, myofibroblastic, and osteogenic phenotypes in response to pathological stimuli. Initial evidence from human tissues shows that sortilin expression is significantly upregulated in calcific aortic valves and co-localizes with areas of active calcification and fibrosis (55). Large-scale genetic studies further support this link, identifying the CELSR2-SORT1 locus as significantly associated with calcific aortic stenosis and aortic valve calcification, with Mendelian randomization suggesting a causal pathway potentially mediated through apolipoprotein B-containing lipoproteins (56).

Mechanistically, sortilin is essential for the pathological transformation of VICs. At the cellular level, single-cell RNA-sequencing analyses have revealed that sortilin is crucial for the emergence of a novel inflammatory myofibroblastic-osteogenic VIC phenotype. This distinct cell cluster, characterized by high co-expression of *SORT1*, *COL1A1*, *IL6*, and *SAA1*, appears to be a key driver of the fibro-calcific process, and its emergence is blunted in the absence of sortilin (55). Functional studies using an aortic valve wire injury model demonstrated that sortilin-deficient mice exhibit significantly reduced valve fibrosis, calcification, and stenosis compared to controls (55). Thus, sortilin transcends the role of a mere biomarker in CAVD; it is a critical pathogenic molecule that integrates inflammatory and fibrotic signals to direct VICs toward a calcifying phenotype, thereby directly fueling valve dysfunction.

In summary, sortilin promotes cardiovascular calcification through distinct yet complementary, tissue-specific mechanisms: in the vasculature, it primarily acts as a vesicular transporter crucial for initiating microcalcification, a process further exacerbated in CKD via carbamylation; in cardiac valves, it drives a fibro-calcific cascade by inducing a pathogenic VIC phenotype. These insights position sortilin not only as a promising biomarker for assessing calcification risk across different cardiovascular beds but also as a compelling therapeutic target for mitigating both vascular and valvular calcification.

## Discussion

Current evidence clearly positions sortilin as an integrative mediator of cardiovascular calcification, exerting influence across metabolic, inflammatory, vascular, and valvular domains. The convergence of clinical observations, genetic associations, molecular studies, and *in vivo* experiments demonstrates that sortilin is not merely a biomarker reflecting disease severity but a causal effector shaping the trajectory of calcification. Its ability links fundamental metabolic disturbances to the cellular machinery that drives osteogenic transformation in both VSMCs and VICs. Importantly, sortilin's role extends beyond EV biogenesis to the orchestration of cytokine secretion, matrix remodeling, autophagy inhibition, and inflammatory amplification, indicating a multifaceted contribution to the fibro-calcific milieu.

Despite these insights, significant questions remain. The context-dependent effects of SORT1 in lipid metabolism raise challenges in predicting systemic consequences of SORT1-targeted therapies. The existence of sex-specific differences in sortilin-driven calcification also suggests underlying hormonal or transcriptional modulators that remain largely unexplored. Additionally, while genetic and mechanistic studies provide compelling evidence for its involvement in CAVD and vascular calcification, it is not yet clear which cellular pools of sortilin—hepatic, vascular, valvular, or immune—are most critical for therapeutic targeting. The development of precise inhibitors will require deeper understanding of sortilin's ligand specificity, domain interactions, and post-translational modifications in disease contexts such as CKD, diabetes, and chronic inflammation.

From a translational perspective, sortilin represents a promising yet complex therapeutic target for cardiovascular calcification. Preclinical evidence is encouraging: genetic Sort1 deficiency or antisense oligonucleotide (ASO)-mediated silencing significantly reduces vascular and valvular calcification in mouse models, supporting the feasibility of sortilin inhibition. Circulating sortilin levels and its modified forms (e.g., carbamylated sortilin) also show potential as biomarkers for early detection or rapid progression of calcification, particularly in patients with chronic kidney disease (CKD). Therapeutically, disrupting sortilin-dependent vesicular trafficking or specific ligand–receptor interactions could attenuate both dyslipidemia-driven and inflammation-mediated calcifying pathways, potentially offering a paradigm-shifting approach that bridges metabolic risk modulation with direct inhibition of local calcification.

Nevertheless, several challenges must be addressed before clinical translation. As a multi-ligand trafficking receptor, sortilin participates in diverse physiological processes, including lipoprotein metabolism, GLUT4 trafficking, neurotensin signaling, progranulin transport, and autophagy. Systemic inhibition may therefore produce context-dependent off-target effects on lipid profiles, insulin sensitivity, neuronal function, or platelet activity. Tissue-specific roles further complicate targeting: while inhibiting vascular/valvular sortilin appears beneficial for calcification, hepatic sortilin exerts complex and sometimes opposing effects on VLDL secretion depending on the metabolic state (normolipidemic vs. obese/ER-stressed). Additionally, sex-specific responses have been observed, with stronger anti-calcific effects of ASO in male mice, possibly related to estrogen-autophagy interactions.

Future development should therefore focus on domain-specific or tissue-restricted modulators—such as cell-type-targeted ASOs, small-molecule inhibitors, or antibodies that selectively block pathogenic ligand interactions—to maximize therapeutic benefit while minimizing risks. Continued research into ligand mapping, tissue-specific functions, and sex differences will be essential to fully unlock the translational potential of sortilin-directed therapies.

## Conclusion

Sortilin sits at the mechanistic crossroads of cardiovascular calcification, integrating metabolic and inflammatory signals into cellular programs that drive vascular and valvular mineralization. Its dual role as a biomarker and pathogenic effector makes SORT1 an attractive, yet complex, therapeutic target. Advancing our understanding of its tissue-specific functions, regulatory mechanisms, and interaction with systemic risk factors will be essential for developing effective interventions. Future translational efforts must aim to harness this knowledge to achieve clinically meaningful prevention or reversal of cardiovascular calcification.
